# Impacts of exoprosthesis use in dogs with partial amputation and limb malformations: a systematic review

**DOI:** 10.3389/fvets.2025.1699152

**Published:** 2025-11-06

**Authors:** Miriã Mamede Noronha de Souza, Jackson Nazareno Gomes de Lima, Danilo José Ayres de Menezes, Lucas Rannier Ribeiro Antonino Carvalho

**Affiliations:** 1 Postgraduate Program in Animal Science and Health, Center for Rural Health and Technology, Federal University of Campina Grande, Patos, Brazil; 2 Department of Physiology and Pharmacology (FYFA), Karolinska Institutet, Stockholm, Sweden

**Keywords:** prosthetics, physical rehabilitation, orthopedics, 3D printing, limb amputation

## Abstract

**Introduction:**

Congenital malformations and limb amputations are common causes of locomotor impairment in dogs, affecting their mobility and well-being. Exoprostheses reemerge as a promising alternative for restoring locomotor function and improving animal welfare. This study aimed to conduct a systematic review of the impacts of exoprostheses used in dogs with limb amputations or malformations.

**Methods:**

The research followed PRISMA guidelines, with searches conducted in PubMed, Web of Science, and SciELO databases, using terms related to dogs, prosthetics, 3D printing, and locomotion. Original articles with clinical applications of socket-type exoprostheses in dogs, made by 3D printing or similar materials, were included. Studies without direct clinical data, implantable prostheses, and assistive devices were excluded. The selection was made independently by two reviewers, and the snowballing strategy broadened the analysis.

**Results:**

After screening and analysis, ten articles were included for synthesis. Among these studies, the main causes of amputation were trauma, congenital deformities, and neoplasms, affecting both thoracic and pelvic limbs. Regarding the exoprosthesis manufacturing approach, most studies used the traditional method of vacuum forming a thermoplastic over a positive form, and three describe the use of 3D printing as an alternative to producing animal prostheses. The studies reported significant improvements in dogs’ mobility and quality of life, although complications such as skin lesions and difficulties with fixation were reported, necessitating adjustments and ongoing veterinary supervision.

**Discussion:**

Customized exoprostheses offer effective and affordable solutions for dogs with amputations, promoting functional improvements and well-being. Further research is warranted to enhance durability and establish standardized protocols for clinical use.

## Introduction

In companion animals, congenital limb malformations, as well as amputations resulting from traumatic injuries or surgical interventions, are a common cause of impaired locomotor function, directly affecting their mobility and quality of life (1, 2). The adoption of socket prostheses, also called exoprostheses, for companion animals has become a growing therapeutic solution in veterinary practice (3).

Exoprosthesis enables the functionality of a partially missing limb, whether due to amputation or congenital deformity, providing a solution for restoring mobility and locomotor function (4–6). These devices have shown more positive results and superior adaptation in dogs with limb amputations, especially in the areas below the carpal and tarsal joints, since these regions offer a larger area for fixation of the prosthesis, allowing better anchorage and stability, facilitating the process of adaptation to the residual limb (4, 5).

Prosthetics developed using additive manufacturing or 3D printing technology enable the creation of highly customized solutions, precisely tailored to the specific needs of each animal, resulting in improved adaptation and functionality. One of the greatest advances enabled by 3D technology is the use of precise patient measurements, combined with 3D scanners and/or computed tomography (CT), to generate a digital model of the prosthesis with extreme accuracy (2, 7, 8). This also involves the use of economically viable methods and materials, which make prosthetic production more accessible and contribute to the stability of dogs’ gait (9, 10).

The effectiveness of adapting to an exoprosthesis in dogs is influenced by multiple factors. The design and material of the device directly affect comfort and functionality (9), while the active involvement of the owner is crucial to support the animal throughout the adaptation process. Rehabilitation plays a key role in restoring strength, coordination, and mobility, as well as facilitating the reintegration of the dog into daily activities, reducing the risk of deformities and joint degeneration, and promoting overall well-being and quality of life (3, 11). According to Lee et al. (12), in veterinary medicine, the pet owner plays a crucial role in the success of treatment and rehabilitation, particularly in cases involving the locomotor system, as they are responsible for tasks such as prosthesis management, physiotherapy, and daily care.

The growing use of exoprostheses and their ability to modify conventional approaches make a detailed evaluation of their efficacy and benefits in treating animal limb pathologies essential (5). In this context, analyzing the effects of exoprostheses in dogs is important to improve knowledge about their more effective application. Understanding the challenges faced during adaptation and clinical outcomes will enable improved treatments, offer more effective alternatives and promoting improved recovery and quality of life for dogs.

The aim of this systematic review is to analyze the impacts of exoprostheses in dogs with amputations or malformations, highlighting the benefits, challenges, and clinical outcomes associated with the adaptation and use of these prostheses.

## Methodology

### Research design

This systematic review evaluated the impacts and benefits of exoprostheses in dogs, with an emphasis on device customization and their effects on animal locomotion. To this end, we used the PRISMA (Preferred Reporting Items for Systematic Reviews and Meta-Analyses) methodological guidelines, adapted from (13), to answer the following question: “What are the impacts and benefits of exoprostheses in dogs?”

The research question was formulated based on the PICO methodology, with the following elements: P: dogs as a population; I: exoprosthesis customization as an intervention; C: comparison between different manufacturing methods; and O: improvement in locomotion as an outcome.

### Sources and search strategies

The bibliographic survey was conducted from March 1 to 5, 2025, through systematic searches in three electronic databases: PubMed, Web of Science, and SciELO. The search was structured by combining controlled and free terms, adjusted for each database, using the following search strategy: (“Dogs” OR “dog” OR “canine” OR “canines”) AND (“Prostheses” OR “3D Printing” OR “3D printed prostheses” OR “additive manufacturing” OR “custom prostheses”) AND (“Locomotion” OR “Gait” OR “Mobility” OR “Biomechanics”).

### Eligibility criteria

This review included studies addressing dogs using socket-type exoprostheses, manufactured by 3D printing or other materials, as well as devices applied to the thoracic or pelvic limbs. Only original articles published in peer-reviewed journals, written in English or Spanish, and with no time limit, were considered.

Studies addressing endoprostheses, exo-endoprostheses, bone-integrated prostheses, or any implantable devices were excluded, as well as those involving exclusively experimental models without direct clinical application. Also excluded were studies addressing exclusively wheelchairs or other mobility assistive devices without prostheses, studies involving animal species other than canines, and those addressing prostheses for other anatomical regions, such as the face or spine, without focusing on the limbs.

### Study selection

After searching for and exporting the articles to the Mendeley® reference manager, duplicates were removed. Two researchers then independently reviewed the titles and abstracts of the identified articles, excluding those that did not meet the eligibility criteria. The selected articles were evaluated in full text, and any disagreements between reviewers were resolved by consensus.

To complement the database search and broaden the scope of the review, a backward snowballing strategy was applied after the initial PRISMA-based selection. In this approach, the reference lists of all included articles (seed articles) were manually screened to identify additional relevant studies that met the predefined inclusion criteria. All newly identified articles were independently evaluated by both reviewers using the same eligibility criteria and consensus procedure adopted in the main selection phase. This process allowed the inclusion of four additional studies, as shown in the PRISMA flow diagram.

### Data extraction and analysis

To collect information, the selected articles were reviewed, and the following data were extracted and organized into a table: authors and year of publication, study location (country), affected limb, cause of partial limb, materials used in manufacture, evaluation and monitoring, and main study conclusions.

## Results

This search identified 200 studies, distributed as follows: 120 in PubMed, 79 in Web of Science, and 1 in SciELO. After removing 54 duplicates, 146 articles remained, which were screened by title and abstract. From this screening, 134 studies were excluded because they did not meet the previously established inclusion criteria. Thus, 12 articles were selected for full reading, but 5 of these did not have the full text located, leaving 7 studies for detailed analysis. Of these, 2 were excluded because they did not present data relevant to the objective of the review, resulting in the initial inclusion of 5 articles.

**Figure 1:**
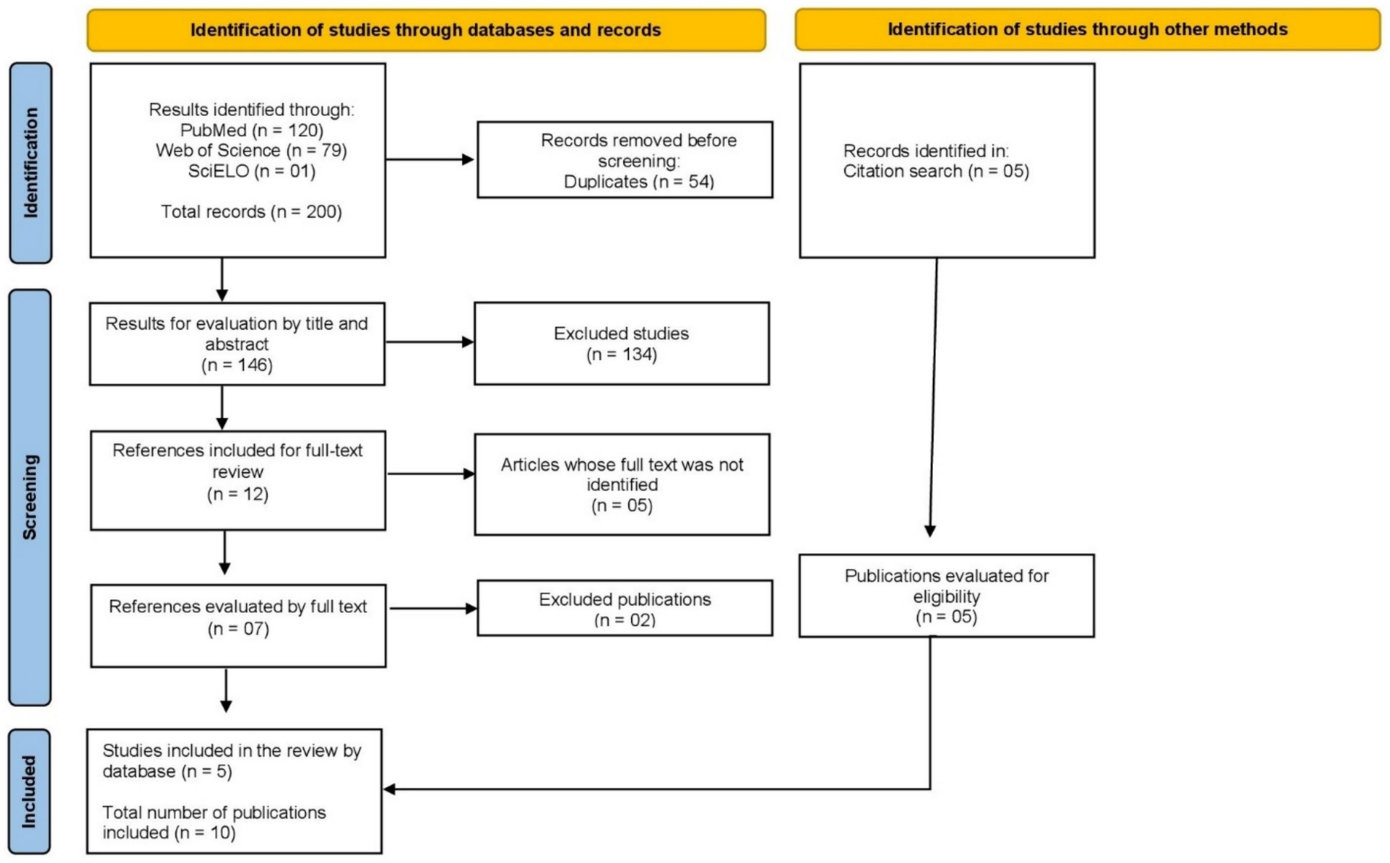
Flow of the process of identification, selection, and inclusion of studies in this systematic review [Adapted from Page et al. (13)].

Given the limited number of eligible studies, reflecting the innovative nature of the topic, a backward snowballing strategy was applied, in which the reference lists of the included articles were screened to identify additional relevant works. This process led to the inclusion of five additional studies, as shown in Figure 1, resulting in a final synthesis of ten articles for this systematic review.

The information considered important to elucidate the guiding question of this review was collected and transferred to the following Table 1.

**Table 1:** Categorized data from the articles included in this systematic review on the use of exoprostheses in dogs.

Author/Year	Country	Limb affected	Cause of partial limb/history	Used material /technique	Evaluation and monitoring	Main conclusions
Adamson et al., 2005 (15)	USA	1 thoracic limb	Congenital deformity (malformation)	Not described	Daily prosthesis use time: 4 h Periodic veterinary evaluations (X-Ray) Total prosthesis use time not specified	The use of the exoprosthesis improved mobility and limb support. Adaptation was positive. Adjustments were necessary, and skin lesions appeared.
Carr et al., 2018 (20)	USA	24 prostheses 12/24 thoracic limbs 12/24 pelvic limbs	Trauma (9) Congenital deformities (9) Neoplasm (4) Infection (1) Unknown (1).	Thermoplastics (not specified) O&P Method* (Animal Ortho Care)	Retrospective research article Time of use and monitoring: Not specified	The use of exoprostheses has demonstrated a positive impact on quality of life, with a high rate of adaptation and improved mobility.
Espín-Lagos et al., 2023 (14)	ECU	1 thoracic limb	Not described	Acrylonitrile Butadiene Styrene (ABS) 3D Printing	Adaptation phase only No monitoring described	The development of the 3D prostheses restored the dog’s autonomy, demonstrating good adaptation, although a period of adjustment was necessary due to the posture acquired after the amputation.
Marcellin-Little et al., 2015 (4)	USA	1 pelvic limb	Not described	Acrylonitrile Butadiene Styrene (ABS) O&P Method*	Not described	Exoprosthesis offers significant benefits for the animal’s mobility and quality of life.
Mendaza- DeCal et al., 2023 (10)	ESP	5 prostheses 3/5 thoracic limbs 2/5 pelvic limbs	Not described	PETG and TPU filament 3D Printing	Monitored tests and periodic visits Time and frequency not specified	The animals showed beneficial results and progressively reduced their lameness by beginning to use the affected limb. The dogs did not participate in rehabilitation programs, and long-term applications were limited.
Phillips et al., 2017 (19)	GBR/AUS	13 prostheses 8/13 thoracic limbs 5/13 pelvic limbs	Congenital deformities (6) Trauma (4) Surgical complications (2) Neoplasm (1)	Polypropylene (PP) O&P Method*	Daily prosthesis use time: 6–8 h Biomechanical testing and owner questionnaires Periodic veterinary evaluations Total prosthesis use time not specified	Fitting a stump and socket prosthesis can be an effective alternative for dogs that are not good candidates for total amputation. The most common complications observed were pressure ulcers and poor tolerance to the prosthesis.
Rosen et al., 2022(6)	USA	7 prostheses (The study does not specify which limb)	Neoplasia – osteosarcoma and soft tissue sarcomas (2) Birth defects (2) Traumatic amputation/amputation secondary to trauma (2) Unknown (1)	Thermoplastics (not specified) O&P Method* (OrthoPets)	Objective gait analysis (OGA) performed at fitting and at 3, 6, and 12 months. Monthly online questionnaires assessed complications and owner perception. Daily prosthesis use time: Not specified Follow-up: 12 months	Variability in the residual limbs of patients who used prosthetics, the lack of a standardized approach to amputations, and the presence of congenital anatomical defects in the patients created a non-uniform analysis. Patient conformation may have introduced a confounding variable into the data analysis.
Souza et al., 2025 (2)	BRA	1 pelvic limb	Traumatic amputation	Polylactic Acid (PLA) 3D Printing	Biomechanical testing and simulations on first use Monitored for 4 weeks	The animal accepted the exoprosthesis well, without any injuries or complications. The use of the prosthesis improved the animal’s mobility and quality of life, positively impacting its routine and well-being.
Wendland et al., 2023(21)	USA	12 animals 10/12 thoracic limbs 2/12 pelvic limbs	Neoplasia (6) Trauma (3) Congenital deformity (1) Recurrent infection (1) Carpal lysis of unknown etiology (1)	Thermoplastics (not specified) O&P Method* (OrthoPets)	Objective gait analysis (OGA) was performed at fitting and at each recheck. Follow-up for at least 6 months with monthly rechecks to monitor prosthesis fit, comfort, and gait. Owners reported prosthesis use 6–7 days per week.	The use of exoprostheses allowed the recovery of quadrupedal gait in most cases. It presented challenges such as suspension difficulties, pressure ulcers, bursitis, infection, prosthesis aversion, and dermatitis.
Wendland et al., 2019(5)	USA	47 prostheses	Trauma (34%) Congenital deformity (21%) Unknown (21%) Neoplasm (17%) Other (7%)	Thermoplastics (not specified) O&P Method* (OrthoPets)	Retrospective research article Daily prosthesis uses time: Median 2–6 h/day Follow-up: medical records and radiographs when available; no standardized prospective rechecks	The high rate of owner satisfaction and the positive clinical results presented by this study.

Data includes information on authors, year of publication, study location, affected limbs, causes of partial limb, materials used, evaluation and monitoring, and main conclusions of each study. *Orthoses and Prostheses Method: Vacuum forming a thermoplastic over a positive form produced on a CNC machine and hand-building.

**Figure 2:**
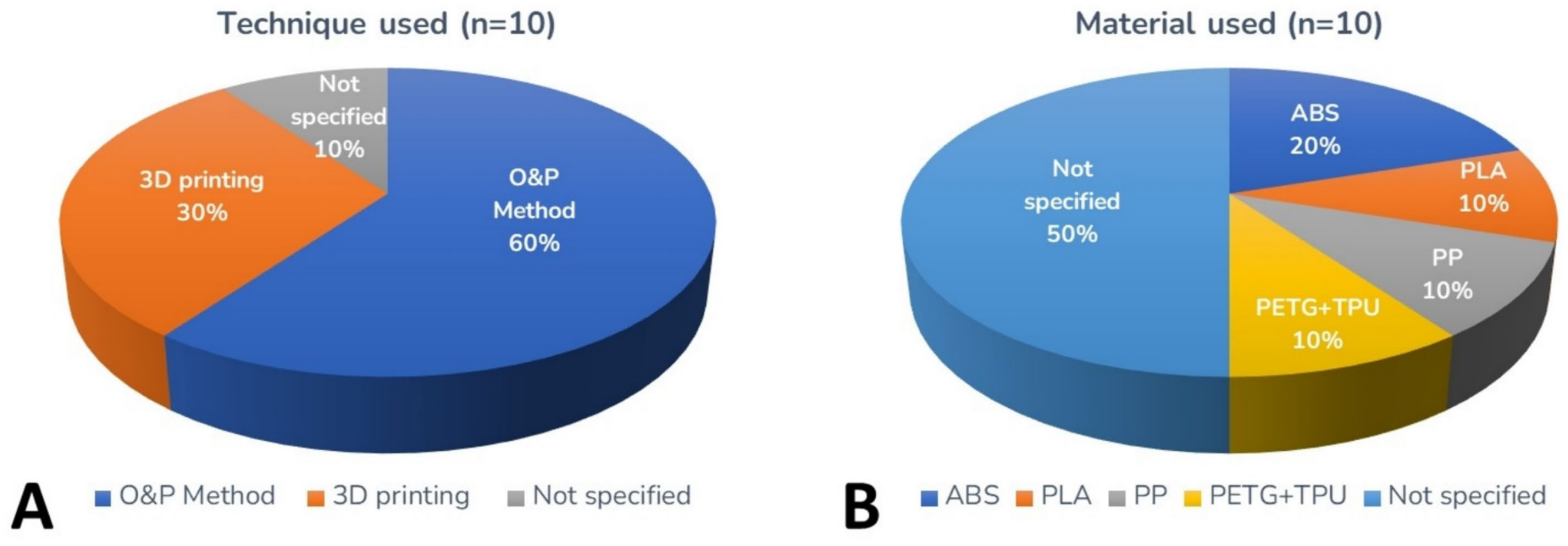
Pie charts referring to the production technique of exoprostheses for dogs **(A)** and material used **(B)**.

In the studies analyzed, amputations occurred primarily due to trauma, congenital deformities, and neoplasms, although the exact frequency of these causes varies between studies. The affected limbs were both thoracic and pelvic, with some studies citing thoracic limb amputations more frequently. The results regarding the type of technique and material used to manufacture the prostheses were described in Figure 2. Only 1 study of 10 did not report the materials used. The remaining 9 articles indicated the use of thermoplastics, of which 5 identified the specific polymers: Acrylonitrile Butadiene Styrene (ABS), Polylactic Acid (PLA), Polypropylene (PP), Polyethylene Terephthalate Glycol Modified (PETG) and Thermoplastic Polyurethane (TPU). Four studies mentioned the use of thermoplastics but did not specify the type (Figure 2). Only 3 of 10 studies (30%) reported the use of 3D printing in the production of prostheses (2, 10, 14), while the majority described devices manufactured through traditional O&P techniques such as vacuum forming and manual fabrication (Figure 2; Table 1). However, there is no consensus on the predominance of any material, and the exact details of the prosthesis’s composition were not fully described in all cases.

In addition to plastic modeling (manual or 3D printing), exoprostheses require complementary materials such as covering and protective structures for the amputated stump, non-slip material for the ground contact area, and in some cases, external fixation structures (2). Therefore, the use of exoprostheses has had a positive impact on dogs’ mobility and quality of life, with favorable adaptation observed in many cases. However, complications such as pressure ulcers, dermatitis, and difficulties in securing the prostheses were reported in some studies. The need for continuous adjustments was mentioned due to variability in residual limbs and the lack of a standardized approach to amputations, which influenced data analysis.

## Discussion and final considerations

Socket prosthetics are still rarely used in veterinary medicine, but demand for them has increased among owners of animals with orthopedic conditions (10). This increased demand demonstrates a greater awareness of available therapeutic alternatives and the desire to provide animals with a better quality of life after amputations or malformations.

An exoprosthesis consists of different components, such as the socket, which fits over the residual limb, ensuring fixation of the prosthesis; the stem or pylon, which serves as the support structure for the device; and the ground contact device, such as an artificial foot, which allows the animal to move with stability (15). In all the studies analyzed, the use of exoprostheses was observed as a solution for the rehabilitation of animals with compromised limbs, highlighting the effectiveness of these devices in promoting locomotion and well-being in dogs.

Prosthetics can be produced by fused deposition molding (FDM) from different filament materials, such as polylactic acid (PLA), polypropylene (PP), polyethylene terephthalate glycol modified (PETG), acrylonitrile styrene acrylate (ASA), nylon, polycarbonate (PC), acrylonitrile butadiene styrene (ABS), and thermoplastic polyurethane (TPU). PLA is the most widely used due to its ease of printing, although it has lower mechanical and thermal resistance, and it is commonly applied in animal prostheses because of its biocompatible nature and low toxicity, which reduces the risk of poisoning if the animal chews or ingests part of the device. ABS stands out for being more robust and durable, better withstanding heat and impact. TPU, in turn, is a flexible elastomer, like rubber, capable of bending and compressing without losing its functional properties (8, 16, 17).

Regarding the production method of exoprostheses, the majority reported manufacturing using traditional thermoplastic modeling methods. 3D printing was applied in only three of the analyzed studies. Even with the growing descriptions of the use of this technology in veterinary medicine — surgical planning, guide construction, custom models, and anatomical studies — applications for prosthetic manufacturing are still less considered, possibly due to limited access to methods, cost-effectiveness, and process feasibility (18).

The most frequently observed causes of amputation were trauma and congenital deformities, followed by neoplasia, which, although present, had a lower occurrence compared to the first two causes. This is similar to what was described by Phillips et al. (19), who observed trauma and congenital anomalies as the most prevalent causes, with a reduced occurrence of neoplasia, which may reflect the greater versatility of stump-fitting prostheses, in addition to the greater likelihood of comorbidities associated with these etiologies.

Assessing the level of limb impairment is necessary in veterinary clinical practice, as it directly influences strategies for preserving the affected limb. In animal amputations, it is often suggested that prostheses be viable up to more distal levels, such as the upper third of the radius/ulna and the mid-tibia (4). Regarding the animals included in the studies, it was observed that most had at least half of the residual limb preserved, which facilitated the integration of the exoprosthesis (2, 4, 5, 14, 15, 19–21). However, Mendaza-DeCal et al. (10) reported the use of exoprostheses in dogs with shorter stumps than recommended, which may compromise stability and comfort during adaptation.

Regarding locomotion, most of the studies evaluated indicated a positive response to the use of exoprostheses, with the animals able to perform daily activities. For example, in the study by Carr et al. (20), of the 24 patients evaluated, 79% (*n* = 19) were able to trot, 70% (*n* = 17) climbed stairs, 54% (*n* = 13) were able to jump over furniture, and 79% (*n* = 19) participated in activities such as playing fetch. Thus, the results show that exoprostheses are important for restoring the functional capacity of animals, allowing them to perform a variety of movements for mobility.

The reviewed studies indicated a high approval rate among owners who chose exoprostheses for their dogs, even in the face of complications. In the study by Wendland et al. (5), most owners interviewed stated that they would choose this treatment option again and would recommend the use of prostheses to other owners. This demonstrated the owners’ confidence in the effectiveness of exoprosthesis, despite the difficulties encountered during the adaptation process. However, this perception can be influenced by subjective factors, as owners often lack a clear benchmark against which to compare the recovery of an animal that underwent only amputation (21).

Another relevant aspect is that veterinarians must carefully consider the impact of the procedure on the animal’s welfare, considering the available data to balance the potential benefits with the risk of complications (21). Therefore, it became important to evaluate the animals’ ability to adapt to the proposed treatment, considering factors such as age, general health, and individual response to the procedure.

When analyzing the complications reported in the studies, it was found that 7 of the 10 studies described some type of adverse effect associated with the use of exoprostheses (5, 6, 14, 15, 19–21). Among the most common problems, skin lesions stood out. Although generally mild, these lesions may require temporary suspension of prosthetic use to allow the skin to recover, interfering with the animal’s adaptation process (5, 6, 15, 21). Furthermore, factors such as improper prosthetic fit, failure to follow the progressive use protocol, and individual patient sensitivity may directly contribute to these complications (6).

Despite the occurrence of these challenges, studies have indicated that, in most cases, it was possible to manage the problems without the need to permanently discontinue exoprosthesis use (6, 21). Measures such as adjustments to the device’s fit and guiding owners through a gradual adaptation period were identified as fundamental strategies to reduce the impact of these challenges and promote better acceptance of the prosthesis by the animal. According to Souza et al. (2), adaptation to the device requires careful and progressive monitoring, in which physical adjustments may be necessary over time. Veterinary supervision combined with the commitment of owners is important to ensure that the animal adapts to the new device comfortably and functionally. Maintaining an adequate exercise routine is also important in the rehabilitation process, as physical activity contributes to the animal’s adaptation to the device, in addition to helping preserve muscles and improve limb functionality (4, 19).

Phillips et al. (19) describe that socket prostheses for thoracic and pelvic limbs have similar complication rates, a finding that aligns with the results of the other six studies analyzed that described complications associated with the use of exoprosthesis. This similarity indicates that the challenges faced by patients are not exclusively related to the type of prosthetic limb, but rather to factors common in all cases, such as prosthesis fit, animal adaptation, and the need for constant monitoring to minimize adverse effects.

Research on exoprostheses for dogs is still limited, with a limited number of studies available in literature. Among the ten studies identified, six were conducted in the United States (4–6, 15, 20, 21), while Brazil (2), Australia and the United Kingdom (19), Spain (10), and Ecuador (14) contributed only one study each. In this context, scientific literature on exoprostheses in veterinary medicine is more concentrated in North America but remains limited. This is a relatively new field that is growing, with technological advances and increased interest in their clinical application. This makes it important to expand this research to improve the use of exoprosthesis in veterinary practice.

A consistent limitation observed across the included studies concerns the lack of standardized outcome evaluation and insufficient reporting of follow-up duration and prosthesis use. In most reports, the type of outcome assessment, whether based on gait observation, owner feedback, or clinical examination, was either subjective or not described in detail. Only a few studies, such as Wendland et al. (21), specified a defined follow-up period and frequency of prosthesis use, providing objective information through observational gait analysis.

Furthermore, although the number of kinematic studies in veterinary medicine is increasing, there are still no established protocols on how to collect kinematic data in canines (22), which contributes to the variability in outcome reporting. This general absence of quantitative evaluation criteria or standardized follow-up intervals hampers the comparability of results and the ability to draw evidence-based conclusions regarding prosthetic efficacy and long-term adaptation. Future studies should adopt more consistent reporting standards, including detailed descriptions of outcome measures, prosthesis use time, and follow-up protocols, to improve methodological transparency and support the advancement of evidence-based veterinary prosthetics.

Finally, to the best of the authors’ knowledge, this is the first systematic review focused specifically on the use of exoprostheses in dogs with amputations. While a review by Kneringer and Schnabl-Feichter (23) compared ITAP and exoprosthetic approaches in veterinary medicine, the present study uniquely synthesizes evidence regarding exoprostheses alone, highlighting their potential as innovative solutions for limb functional recovery. Although few studies have focused on the application of 3D technology for this purpose, it presents a promising alternative for overcoming complications associated with the variability of residual limbs and the lack of standardization in amputations. However, more research is still needed to evaluate the long-term durability and performance of these prostheses. This review provides a foundation for future investigations aimed at improving exoprosthesis design, addressing associated complications, and maximizing clinical benefits for dogs and their owners.

## Data Availability

The original contributions presented in the study are included in the article/supplementary material, further inquiries can be directed to the corresponding author.
